# Ostéchondromatose synoviale de l'articulation carpo-métacarpienne du pouce chez une patiente atteinte d'un lupus érythémateux systémique

**DOI:** 10.11604/pamj.2015.22.185.8065

**Published:** 2015-10-23

**Authors:** Hanen Loukil, Faten Frikha, Mouna Snoussi, Saida Garbaa, Raida Ben Salah, Zouhir Bahloul

**Affiliations:** 1 Service de Médecine Interne, CHU Hédi Chaker, Sfax, Tunisie

**Keywords:** Ostéochondromatose, lupus, arthrose, osteochondromatosis, lupus, arthrosis

## Abstract

L'ostéchondromatose synoviale est une métaplasie du tissu synovial. Elle engendre de petites masses cartilagineuses qui font saillie à la face interne de la synoviale, puis, se pédiculisent et enfin s'en détachent en développant des chondromes libres. On en distingue deux formes d'ostéchondromatose, la forme primitive, qui est rare, et la forme secondaire plus fréquente dont l'origine est généralement l'arthrose. Nous rapportons un cas d'ostéchondromatose synoviale de l'articulation carpo-métacarpienne du pouce chez une patiente atteinte d'un lupus érythémateux systémique.

## Introduction

L'ostéchondromatose synoviale est une métaplasie du tissu synovial. Elle engendre de petites masses cartilagineuses qui font saillie à la face interne de la synoviale, puis, se pédiculisent et enfin s'en détachent en développant des chondromes libres. Ces derniers peuvent s'ossifier et justifier ainsi le terme d'ostéchondromatose [1–3]. On en distingue deux formes, une forme primitive, et une forme secondaire dont l'origine est généralement l'arthrose. L'ostéochonromatose au niveau de la main est décrite comme rare [2]. Nous rapportons un cas d'ostéchondromatose synoviale de l'articulation carpo-métacarpienne du pouce chez une patiente atteinte d'un lupus érythémateux systémique (LES).

## Patient et observation

**Figure 1:**
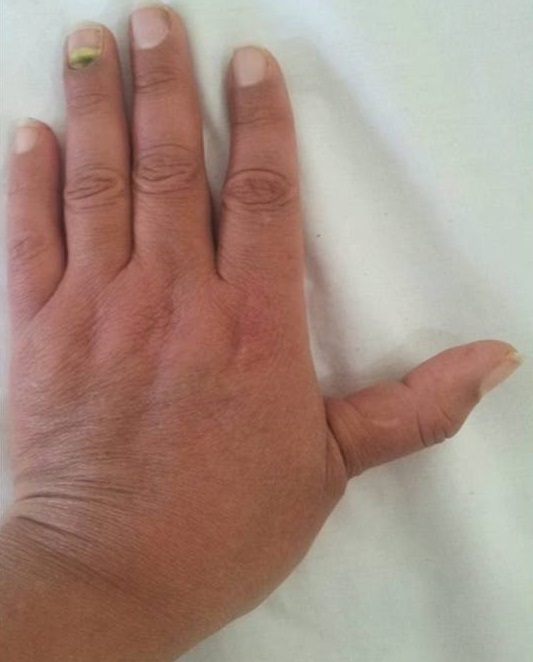
Main gauche: aspect de pouce adductus

**Figure 2:**
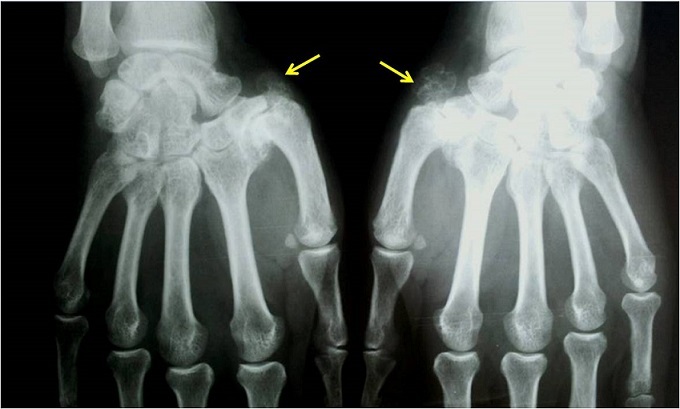
Radiographie standard des 2 mains: multiples formations de petites masses arrondies de tissu cartilagineux réalisant l'aspect en en chou-fleur

Une femme âgée de 48 ans a été hospitalisée dans notre service en 2012 pour une polyarthrite touchant de façon bilatérale les grosses et les petites articulations. Le diagnostic de LES dans sa forme bénigne a été retenu devant la notion de photosensibilité, l'atteinte articulaire, et un bilan immunologique positif (anticorps antinucléaires positifs à 1/1280 et anti-ADN natifs positifs). Elle a été traitée par une corticothérapie associée à l'hydroxychloroquine avec une bonne évolution. Deux ans plus tard, elle se présentait avec des douleurs mécaniques des 2 pouces. L'examen clinique objectivait une saillie de la tète métacarpienne des 2 pouces avec une déformation en « pouce adductus » bilatérale plus accentuée à gauche (Figure 1). La radiographie standards des 2 mains objectivait une rhizarthrose évoluée avec volumineuses productions ostéophytiques en regard de l′articulation trapézo-métacarpienne évoquant une ostéchondromatose multiple (Figure 2). Sous traitement symptomatique antalgique et une orthèse des 2 pouces, la malade a été stabilisée.

## Discussion

L'ostéochondromatose est une pathologie dont la définition la plus communément adoptée actuellement est celle proposée par Jaffe en 1958 et reprise par Roderer en 1981: « métaplasie au sein du tissu synovial de certaines cellules en chondrocytes qui entrainent la formation de petites masses arrondies de tissu cartilagineux, qui font saillie à la face interne de la synoviale, puis se pédiculisent et enfin s'en détachent, devenant des chondromes libres intra-articulaires » [1]. Il en résulte une gêne à la mobilisation et un syndrome tumoral aboutissant à plus ou moins long terme, en l'absence de traitement, à une destruction articulaire.

La fréquence globale de cette affection est probablement sous-estimée dans la population mondiale; elle atteint le plus souvent les grosses articulations et peu de cas d'atteinte de la main et des doigts ont été publiés [2–5]. Milgram [6] distingue trois phases dans le cycle de l'ostéchondromatose: Phase 1: maladie intrasynoviale sans corps étrangers libres; Phase 2: prolifération synoviale active avec corps étrangers libres; Phase 3: corps ostéochondromateux libres multiples sans maladie intrasynoviale.

Le diagnostic radiologique précoce de l'ostéochondromatose n'est possible qu’à partir du stade 2 (de Milgram) avec corps étrangers. Les radiographies standards montrent des chondromes calcifiés dans l'aire para-articulaire. Cependant, le scanner ou mieux encore l'IRM trouvent leur place dans la chondromatose pure radiotransparente [7]. On en distingue deux formes d'ostéchondromatose, la forme primitive, qui est rare, et la forme secondaire plus fréquente dont l'origine est généralement l'arthrose. D'autres affections, comme, la dysplasie épiphysaire, la maladie de Legg-Perthes Calve, l'ostéonecrose aseptique, la polyarthrite rhumatoïde, le diabète, la chondrocalcinose, ont été citées comme des causes possibles. Dans le cas de notre patiente, nous attribuons le développement de l'ostéchondromatose au lupus. A notre connaissance, C'est le deuxième cas d'ostéchondromatose associée au lupus [8].

Le traitement de l'ostéchondromatose diffère selon sa forme primaire ou secondaire. Dans les formes primaires, on préconise une synovectomie avec ablation des corps étrangers mais les récidives sont fréquentes. Dans les formes secondaires, l’évolution est le plus souvent lente et le traitement est étiologique par traitement de l'arthropathie préexistante et ablation des corps étranger.

La revue de la littérature montre que ces lésions évoluent le plus souvent avec bénignité et le pronostic peut être, avec Sim [9] et Lichenstein [10] considéré comme bénin dans le ostéchondromatose de la main puisque aucun cas de transformation maligne n′ayant été décrit. Et même si à l′examen microscopique on observe une hyperactivité nucléaire des cellules cartilagineuses, leur multiplication rapide n′est que le témoin d′une activité propre aux cellules cartilagineuses en croissance. La transformation sarcomateuse a été observée dans les chondromatoses au genou et à la hanche. En revanche, les récidives ne sont pas exceptionnelles.

## Conclusion

Cette observation d'ostéochondromatose est particulière du fait de la localisation bilatérale de cette métaplasie et du fait de son association à une maladie lupique.
